# Assessment of Antidepressant Effect of the Aerial Parts of *Micromeria myrtifolia* Boiss. & Hohen on Mice

**DOI:** 10.3390/molecules24101869

**Published:** 2019-05-15

**Authors:** Esra Küpeli Akkol, Fatma Tuğçe Gürağaç Dereli, Mert Ilhan

**Affiliations:** 1 Department of Pharmacognosy, Faculty of Pharmacy, Gazi University, Etiler, Ankara 6330, Turkey; ecztugceguragac@gmail.com; 2 Department of Pharmacognosy, Faculty of Pharmacy, Van Yüzüncü Yıl University, Tuşba/Van 65080, Turkey; eczmertilhan@gmail.com

**Keywords:** antidepressant, forced swimming test, lamiaceae, *Micromeria myrtifolia*, monoamine oxidase, tail suspension test

## Abstract

The currently available antidepressant agents necessitate the development of newer alternatives because of their serious adverse effects and costs. Traditional medicinal knowledge is likely the key that opens the door to discover new medicines. In Turkish folk medicine, the infusion prepared from aerial parts of *Micromeria myrtifolia* Boiss. & Hohen is used as pleasure and medicinal tea for its relaxing action. The present research was conceived to confirm the antidepressant’s potential of this traditional medicinal plant. In this process, first of all, the collected and shade-dried aerial parts of *M. myrtifolia* were powdered and then, extracted using solvents with different polarity as follows; *n*-hexane, ethyl acetate (EtOAc), and methanol (MeOH). The antidepressant activity of the extracts was evaluated by using several in vivo and in vitro experimental models of depression. When the data obtained from the control and experimental groups were compared, it was determined that the MeOH extract was the most active. The active components of this extract were isolated and identified utilizing various chromatographic separation techniques. The MeOH extract was applied to reversed phase (RP-18) column chromatography to obtain five main fractions and they were tested on antidepressant activity models. The isolated compounds from the obtained fractions were elucidated as rosmarinic acid (**1**), myricetin (**2**), apigenin (**3**), and naringenin (**4**) which were assumed to be responsible for the antidepressant activity of the aerial parts. According to the results, rosmarinic acid, myricetin, apigenin, and naringenin showed statistically significant activity on forced swimming test and tetrabenazine-induced ptosis models, whereas only rosmarinic acid showed statistically significant activity on the tail suspension test. Apigenin displayed the highest inhibitory activity on MAO A and B enzymes. Studies in the future should be performed to investigate the antidepressant activity mechanism of these natural compounds. The current research could be an important step in the development of the new agents that can be used in the treatment of depression.

## 1. Introduction

Depression is a widespread chronic psychiatric complaint which interferes with social life and work performance. Today, millions of people of all ages suffer from this disease [1]. According to figures from the World Health Organization, by the year 2020, depression is estimated to be the second known cause of world disability [2], and through 2030, it will probably make the greatest contribution to the burden of disease [3].

Depression is a mental disease associated with the interaction of psychological, social and biological factors. The development of this mood disorder can be triggered by several etiological factors, including personal and environmental conditions, genetic and biochemical parameters [4]. Differences in brain chemicals, low self-confidence personality, and stressful life conditions may contribute to the onset of depression. It is characterized by several symptoms such as the feeling of hopelessness or worthlessness; changes in appetite, weight and sleep patterns; fatigue; increased agitation and decreased interest in pleasurable stimuli. Actually, the most dangerous one is recurrent suicidal thoughts [5,6]. Depression can cause some damages to physical health. Depression-related physical illnesses such as diabetes, obesity [7], cardiovascular diseases [8], stroke, cancer, lung diseases [9] and loss of hearing and vision [10] show an enigmatic mind–body connection. The levels of monoamine neurotransmitters namely serotonin, noradrenaline, and dopamine; corticotrophin-releasing factor; corticotrophin-releasing hormone; cortisol and adrenocorticotropic hormone, and also the actions of hypothalamic–pituitary–adrenal (HPA) axis, adenylyl cyclase, and monoamine oxidase (MAO) are main biomarkers for diagnosis [11].

Medications available for the treatment of depression such as tricyclic antidepressants, monoamine oxidase inhibitors, selective serotonin reuptake inhibitors and specific serotonin-noradrenaline reuptake inhibitors [12]. However, the side profile effect profiles of above mentioned drugs regarding libido, sleep, body weight, and cardiovascular system because the treatment of depression is still not at the desired level [13,14,15]. The search for new alternatives to manage depression is turning scientists into natural resources especially phytochemicals. Large number of plants are traditionally used worldwide by indigenous people for the treatment of neurological health problems, and *M. myrtifolia* is only one of them [16].

Today, a large number of efficient treatment options are available for depression, but the reality is that no perfect solution exists that works quickly and is free of adverse reactions. Therefore, researchers are seeking alternatives to produce more specific, newer, safer, and cheaper medications nowadays and traditional medicinal plants provide an extensive research area for them. Considering traditional uses, thousands of promising plants for the future have not yet been studied in terms of medical potential. This is particularly important for diseases such as depression which have not yet reached the desired therapeutic level.

*Micromeria* is a large genus belongs to Lamiaceae family and widespread in Mediterranean regions. Flora of Turkey contains 14 *Micromeria* species (22 taxa), 12 of which are endemic [17]. *Micromeria* is used traditionally for ages in the cure of inflammation, fever, asthma, skin diseases, cardiac problems and digestive system disorders [18]. Dozens of studies have confirmed the biological activities of *Micromeria* species such as antifungal [19], antimicrobial [20], antioxidative [21], anticholinesterase [22], anti-inflammatory and gastroprotective [23], hepatoprotective [24], cytotoxic [25]. Phytochemical investigations on this genus have indicated the presence of several flavonoid compounds, saponins, tannins, anthraquinones and essential oils [26,27,28,29,30].

One of the *Micromeria* species, *M. myrtifolia*, a perennial suffruticose plant, has been used as pleasure and medicinal tea in Turkish traditional healing system for the treatment of gallstones and gastrointestinal disorders [16,17]. Moreover, the infusion prepared from the aerial parts of *M. myrtifolia* is used as relaxant and sedative by drinking one teacup 1–2 times a day [16,31]. The phytochemical screening of the volatile oil of *M. myrtifolia* indicated that it contained some bioactive constituents; monoterpene hydrocarbons (α- and β-pinene, p-cymene, limonene etc.), oxygenated monoterpene derivatives (such as linalool, camphor, borneol), sesquiterpene hydrocarbons (germacrene D, β-caryophyllene, α-humulene etc.), oxygenated sesquiterpenes (such as caryophyllene oxide), phenolic compounds (thymol, carvacrol, eugenol etc.), fatty acids and derivatives (such as pentadecanoic acid, hexadecanoic acid), carbonylic compounds (nonanal, decanal etc.), hydrocarbons and other types compounds ((*Z*)-Phytol) [32]. There are few studies on the biological activity of the plant [32,33] and to date, no studies have been designed to investigate its antidepressant effect.

Therefore, the aim of the present research is not only to demonstrate the antidepressant-like activity of *M. myrtifolia* in different experimental models of depression in mice, but also to reveal the responsible bioactive constituents of this relaxant ethnomedicinal plant. 

## 2. Results

### 2.1. Biological Activity Studies

In the forced swimming test (FST), the inactivity seen in mice exhibits the behavioral despair seen in humans and conventional antidepressant drugs make a reduction in the duration of inactivity time [34]. If the animal displayed locomotor activity in stressful conditions, it was thought as a positive response for the avoiding of sedation. The results of FST were summarized in Table 1. The MeOH extract and Fractions B and C at the doses of 100 mg/kg shortened the immobility duration significantly with the values of 38.39%, 39.24%, 43.31%, respectively. In addition, rosmarinic acid, myricetin, apigenin and naringenin at the doses of 25 mg/kg significantly decreased the immobility duration with the values of 37.59%, 38.41%, 31.13%, and 28.80%, respectively. Despite this, the EtOAc extract showed a noteworthy activity and the *n*-hexane extract did not exert antidepressant activity in this in vivo model. 

Similarly, TST is based on the observation of inactivity in stressful conditions [35]. Antidepressant drugs have the ability to decrease the duration of inactivity time in this experimental model [36]. As shown in Table 2, the immobility time in TST remarkably decreased after the treatment with the MeOH extract, similar to the reference medicament fluoxetine. The MeOH extract and Fraction B at the doses of 100 mg/kg shortened the immobility time significantly with the values of 35.24%, 36.69%, respectively. Though rosmarinic acid significantly decreased immobility time with the value of 45.65%, the other isolated compounds did not show any remarkable activity in TST model.

According to the monoamine hypothesis of depression, the mechanism of the pathophysiology of depression is the depletion of monoamine neurotransmitters, including serotonin, norepinephrine and/or dopamine in the central nervous system (CNS) [37]. Tetrabenazine, synthetic benzylquinolizine derivative [38], causes depletion of dopamine and other monoamines in the CNS and increases the risk of depressive disorders [39]. Similar results were received in the antagonism of ptosis and hypothermia induced by tetrabenazine test, as shown in Table 3. The MeOH extract antagonized hypothermia, ptosis, and motor depression in mice. However, the rest of the extracts did not show remarkable activity. 

The MAO inhibition assay is a practical and speed test for assessing the inhibition of MAO enzyme, which is known to play an active role in the pathogenesis of depression [33]. In MAO inhibition assay, the MeOH extract inhibited MAO-A and MAO-B enzymes with the IC_50_ values of 4.7 and 1.4, respectively (Table 4).

The MeOH extract was subjected to RP-18 column chromatography to isolate the constituents responsible for the antidepressant activity, and the eluted fractions were grouped into five categories based on their chemical fingerprinting on TLC examination. In the in vivo and in vitro activity studies, Fraction B and C showed a significant activity (Table 1, Table 2, Table 3 and Table 4).

**Figure 1:**
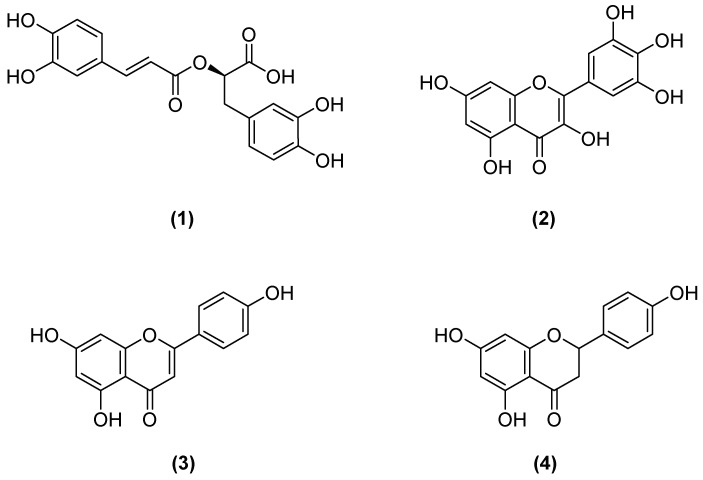
Chemical structures of rosmarinic acid (**1**), myricetin (**2**), apigenin (**3**), and naringenin (**4**) isolated from *Micromeria myrtifolia*.

Thus, Fractions B and C were further fractionated using Sephadex LH-20 and silica jel column. Further, rosmarinic acid (**1**) from Fraction B and myricetin (**2**), apigenin (**3**) and naringenin (**4**) from Fraction C were isolated and were identified using spectral analysis (Figure 1). 

In further studies, isolated compounds were purchased and their antidepressant activities were evaluated. According to the results, rosmarinic acid, myricetin, apigenin, and naringenin showed statistically significant activity on the forced swimming test and tetrabenazine-induced ptosis models, whereas only rosmarinic acid showed statistically significant activity on the tail suspension test (Table 2). Fractions B and C significantly inhibited MAO A and B enzymes. Apigenin isolated from Fraction C displayed the highest inhibitory activity on both enzymes.

### 2.2. NMR Data of Isolated Compounds

*Rosmarinic acid* (**1**): ESI-MS *m*/*z*: 361.0914. ^1^H-NMR (500 MHz, CD_3_OD): δ 7.48 (1H, d, *J* = 15.2 Hz, H-7′), 7.00 (1H, d, *J* = 1.8 Hz, H-2′), 6.88 (1H, dd, *J* = 8.0/1.8 Hz, H-6′), 6.71 (1H, d, *J* = 8.0 Hz, H-5′), 6.21 (1H, d, *J* = 15.2 Hz, H-8′), 6.71 (1H, d, *J* = 1.8 Hz, H-2), 6.61 (1H, d, *J* = 8.0 Hz, H-5), 6.59 (1H, dd, *J* = 8.0/1.8 Hz, H-6), 5.03 (1H, dd, *J* = 9.1/3.5 Hz, H-8), 3.01 (1H, dd, *J* = 14.0/3.5 Hz, H-7b), 2.90 (1H, dd, *J* = 14.0/9.1 Hz, H-7a); ^13^C-NMR (125 MHz, CD_3_OD): δ 168.2 (C-9′), 149.5 (C-4′), 147.3 (C-7′), 146.7 (C-3′), 127.1 (C-1′), 122.6 (C-6′), 116.1 (C-5′), 114.8 (C-8′), 113.8 (C-2′), 167.0 (C-9), 145.7 (C-3), 144.8 (C-4), 129.3 (C-1), 121.3 (C-6), 117.1 (C-2), 116.0 (C-5), 76.0 (C-8), 37.3 (C-7).

*Myricetin* (**2**): ESI-MS *m*/*z*: 319.0448. ^1^H-NMR (500 MHz, CD_3_OD): δ 7.21 (2H, s, H-2′/6′), 6.35 (1H, d, *J* = 1.8 Hz, H-8), 6.13 (1H, d, *J* = 1.8 Hz, H-6); ^13^C-NMR (125 MHz, CD_3_OD): δ 173.1 (C-4), 163.8 (C-7), 160.5 (C-5), 155.6 (C-9), 146.3 (C-2), 145.3 (C-3′/5′), 135.4 (C-3), 135.3 (C-4′), 120.5 (C-1′), 106.1 (C-2′/6′), 104.6 (C-10), 97.7 (C-6), 92.9 (C-8).

*Apigenin* (**3**): ESI-MS *m*/*z*: 271.0596. ^1^H-NMR (500 MHz, CD_3_OD): δ 7.88 (2H, d, *J* = 8.8 Hz, H-2′/6′), 6.91 (2H, *J* = 8.8 Hz, H-3′/5′), 6.73 (1H, s, H-3), 6.42 (1H, d, *J* = 2.0 Hz, H-8), 6.11 (1H, d, *J* = 2.0 Hz, H-6); ^13^C-NMR (125 MHz, CD_3_OD): δ 183.7 (C-4), 166.3 (C-7), 165.8 (C-2), 163.2 (C-5), 163.1 (C-4′), 158.8 (C-9), 131.3 (C-2′/6′), 123.2 (C-1′), 116.0 (C-3′/5′), 105.1 (C-10), 103.9 (C-3), 100.1 (C-6), 94.6 (C-8).

*Naringenin* (**4**): ESI-MS *m*/*z*: 273.0752 ^1^H-NMR (500 MHz, CD_3_OD): δ 7.34 (2H, d, *J* = 8.8 Hz, H-2′/6′), 6.87 (2H, d, *J* = 8.8 Hz, H-3′/5′), 5.92 (1H, d, *J* = 2.2 Hz, H-6), 5.90 (1H, d, *J* = 2.2 Hz, H-8), 5.31 (1H, dd, *J* = 13.1/2.8 Hz, H-2), 3.11 (1H, dd, *J* = 17.8/13.1 Hz, H-3a), 2.68 (1H, dd, *J* = 17.8/2.8 Hz, H-3b); ^13^C-NMR (125 MHz, CD_3_OD): δ 197.3 (C-4), 168.1 (C-9), 165.8 (C-5), 164.5 (C-7), 158.1 (C-4′), 129.5 (C-1′), 128.4 (C-2′/6′), 115.6 (C-3′/5′), 101.8 (C-10), 97.3 (C-6), 96.9 (C-8), 78.5 (C-2), 42.2 (C-3).

## 3. Discussion

Until now, the antidepressant activity of many phytochemical compounds has been investigated. The phenolic compounds, which are among these phytochemicals, were divided into several sub-groups, including phenolic acids, flavonoids, chromones, coumarins, anthraquinones, stilbenes and lignans, based on the changes in their chemical structures [40]. Polyphenols with potent antioxidant properties are suggested for the treatment of different conditions, including degenerative, cardiovascular and gastrointestinal disorders [40,41]. Flavonoid type compounds are generally known for their hepatoprotective, antioxidant, antibacterial, anti-inflammatory and antiviral activities [42,43]. They are also widely studied phytochemicals in terms of their antidepressant activities and mechanisms. The mechanisms at this point could be gene regulation for neurotransmitter receptor expression and reversal of monoamine neurotransmitter attenuations by norepinephrine (NE), dopamine (DA), serotonin (5-HT), and 5-Hydroxyindoleacetic acid (5-HIAA) [44,45,46,47,48]. For example, baicalein exerted antidepressant-like activity by increasing extracellular signal-regulated kinase (ERK) phosphorylation and brain-derived neurotrophic factor (BDNF) [49]; baicalin by decreasing MAO-A and B activity [50]; hesperidin by effecting 5-HT receptors and κ-opioid [51]; quercetin by showing neuroprotective effect through microglial inhibitory pathway [52]; rutin and quercitrin by increasing mRNA expression of pro-opiomelanocortin (a precursor of several types of peptides that seems to be dysregulated in depression) and elevating 5-HT and NE availability in synaptic cleft [53,54].

Plenty of studies have been designed to assess the antidepressant-like activity of apigenin so far. Nakazawa et al. (2003) reported that apigenin, which is a plant-derived flavonoid abundant in fruits of Citrus, possessed antidepressant-like effect in mice and decreased the duration of immobility at the acute doses of 12.5 and 25 mg/kg (i.p.) preventing the stress-induced changes in dopamine turnover in hypothalamus [55]. However, Yi et al. found that acute oral treatment with apigenin did not show any antidepressant-like activity in mice. For this reason, they also tried subchronic and chronic treatment and reported that a two-week oral apigenin treatment at the daily doses of 10 and 20 mg/kg exerted significant antidepressant activity in mice by downregulation of the cAMP pathway and normalization of the central monoamine neurotransmitter systems and HPA axis alterations [56]. Li et al. investigated the antidepressant-like activity of chronic intraperitoneal administration of apigenin and found that it reversed the depressive-like behavior induced by tumor necrosis factor-α (TNF-α) without altering locomotor activity at a dose of 50 mg/kg [57]. As reported by Weng et al., the oral apigenin treatment at the doses of 20 and 40 mg/kg for 21 days possessed significant antidepressant activity in chronic corticosteroid-treated mice by up-regulation of BDNF levels in the hippocampus [58]. Myricetin is one of the well-known flavonols investigated for antidepressant activity. Ma et al. revealed that chronic administration of myricetin at the dosage of 50 mg/kg showed antidepressant effect improving the activity of glutathione peroxidase (GSH-PX) in the hippocampus, and normalizing of the decreased brain-derived neurotrophic factor (BDNF) levels in mice exposed to repeated stress [59]. Several previous studies verified the antidepressant activity of naringenin, which is a dietary a flavanone widely found in peels of citrus fruits, in behavioral models of depression. Yi et al. evaluated neuropharmacological mechanism of naringenin and concluded that naringenin possessed potent antidepressant-like property through neuroprotective and monoamine oxidase inhibitory activities at the doses of 10, 20, and 50 mg/kg in mice [60]. Yi et al. attributed the antidepressant activity of naringenin to elevate the 5-HT, NE, and BDNF levels as well as glucocorticoid receptors [60,61,62]. Recent publications demonstrated that rosmarinic acid is one of the polyphenolic compounds endowed with antidepressant-like effect. Ito et al. attributed the activity of rosmarinic acid (1.0, 2.0, 4.0 mg/kg/bw, i.p.) promotion of neurogenesis in hippocampus of mice [63]. Jin et al. proved that rosmarinic acid administration (daily 5 and 10 mg/kg, for 14 days) showed activity in rats via increasing astrocytic BDNF expression in the hippocampus through modulation of the extracellular-regulated kinase phosphorylation [64]. Kondo et al. suggested that rosmarinic acid (5 and 10 mg/kg/day, for 7 days) exerted antidepressant activity in mice via modulation of dopamine and corticosterone, through the upregulation of tyrosine hydroxylase (TH) gene expression in the animals’ brains [65].

Petersen and Simmonds (2003) reported that rosmarinic acid showed a very low toxicity with a LD50 in mice of 561 mg/kg after intravenous application [66]. Semwal et al. (2016) exhibited that intraperitoneal administration of myricetin at a dose of 1000 mg/kg to mice did not reveal any toxic effects or fatalities. The compound did not cause any toxicity at doses above 100 mg/kg (LD50 value) in zebrafish larvae induced by UVB-generated ROS [67]. Osigwe et al. (2017) reported to possess apigenin very low toxicity [68].

The present study exerted that the MeOH extract of *M. myrtifolia* and its phenolic compounds showed remarkable in vivo and in vitro antidepressant activities. In the discovery of better agents against depression, this work is a step and further studies should be conducted for the determination of the mechanism.

## 4. Materials and Methods

### 4.1. Plant Material

Fresh aerial parts of *M. myrtifolia* were collected from Çıralı-Antalya, Turkey, in April 2016. The voucher sample (GUEF 3487) was deposited at the Herbarium of the Faculty of Pharmacy, Gazi University, Ankara, Turkey. The botanical identification of the plant was realized by Prof. Dr. Hayri DUMAN of Gazi University, Department of Biology, Faculty of Science and Art, Ankara.

### 4.2. Extraction, Fractionation, and Isolation Process

The dried and ground plant materials were subjected to maceration using *n*-hexane, ethyl acetate (EtOAc), and methanol (MeOH) as solvents, respectively at room temperature. Following the filtration process, the prepared extracts were evaporated to dryness through rotary evaporator. The percentage yield of the methanolic extract (17.13%) was higher than *n*-hexane (9.05%) and EtOAc (6.24%) extracts.

#### 4.2.1. Fractionation of MeOH Extract

MeOH extract of the aerial parts (25 g) was subjected to RP-18 column vacuum liquid chromatography 2 L H2O, 1 L H2O:MeOH (90:10), 1 L H2O:MeOH (80:20), 2 L H2O:MeOH (70:30), 2 L H2O:MeOH (60:40), 2 L H2O:MeOH (50:50), 2 L H2O:MeOH (40:60), 2 L H2O:MeOH (30:70), 2 L H2O:MeOH (20:80), 1 L H2O:MeOH (10:90), 1 L MeOH, and 1 L acetone] to obtain 30 fractions, which were combined as follows after thin-layer chromatography (TLC) control using EtOAc:CHCl3:MeOH:H2O (6:4:4:1) as the mobile phase: Fractions A–E. These five different types fractions were tested for antidepressant-like activity using several in vivo model. The results showed that Fraction B and C were more active from others, and then these fractions were chromatographed over Sephadex LH-20 column to obtain pure compounds.

#### 4.2.2. Determination of the Structure of Compounds

Nuclear magnetic resonance (^1^H- and ^13^C-NMR) and mass spectroscopy (MS) techniques were used for the structural elucidation of the compounds. NMR spectra were recorded on a Bruker spectrometer (400 MHz for ^1^H-NMR and 100 MHz for ^13^C-NMR) instrument and using CD_3_OD as the solvent. ESI-MS analyses were performed using a spectrometer (Waters LCT Premier XE UPLC/TOF-MS, Santa Clara, CA, USA). The isolates were identified as rosmarinic acid (**1**), myricetin (**2**), apigenin (**3**), and naringenin (**4**) by assessing and comparing their spectroscopic data with those published in related references. 

### 4.3. Biological Activity Tests

#### 4.3.1. Animals

The experiments were conducted on male mice of BALB/c strain (25–30 g) for the in vivo behavioural experiments and Sprague–Dawley male rats (180–200 g) were used for the MAO inhibitory activity tests. All animals were provided from Kobay Test Animals Laboratory (Ankara, Turkey) and kept in 12 h light/dark cycle at room conditions with free access to laboratory food and water tap ad libitum for three days before the pharmacological experiments. All of the experiments were performed according to the international guidelines on animal experiments and biodiversity rights. Seven mice were used for each experimental group. All the studies were performed conferring to the international rules regarding the animal experiments and biodiversity rights (Kobay Ethical Council Project Number: 255).

#### 4.3.2. Preparation of Test Samples for Bioassay

All of the extracts were administered at the dose of 100 mg/kg in 0.5% aqueous carboxymethyl cellulose (CMC) suspension. The mice in the control group were treated with only 0.5% CMC suspension (vehicle) under the same conditions. Tricyclic antidepressant imipramin at the dosages of 30 and 50 mg/kg (Merck) and fluoxetine at the dosage of 25 mg/kg (Merck) in 0.5% CMC were used as reference medicaments.

#### 4.3.3. Forced Swimming Test (Behavioral Despair Test)

Antidepressant-like activity of each test samples were assessed in the forced swimming test (FST) based on the methodology described by Porsolt et al. [33] with minor modifications. One hour after the oral application of the test samples/reference medicaments, the animals were individually placed in a 20 cm high transparent glass beaker filled with tap water at 23 ± 2 °C to a height of 10 cm and forced to swim. The water was refreshed after each experiment and each mouse was used just once. All of the experiments were videotaped, and the total duration of inactivity (s) was chronometered the last 2 min of the 6 min-long period. Animals were accepted inactive when they did not try to escape, except for movements necessary to hold their heads in the water. Five different experimental groups were formed for the evaluation of the effect of each extract and seven experimental groups were used to assess the potential of the fractions [69].

#### 4.3.4. Tail Suspension Test

The tail suspension test (TST) was performed as described by Steru et al. [70]. One hour after the application of the test materials/reference medicament orally, the mice were individually suspended 50 cm above the floor approximately by 1 cm from the tip of the tail with an adhesive tape. All of the experiments were videotaped, and the total duration of inactivity (s) was scored for the last 6 min of the 10 min-long sessions. Immobility was measured when the mice did not make any struggle to get rid of the tape for at least 1 min. Four different experimental groups were formed for the evaluation of the effect of each extract and six experimental groups were used to assess the activity of the fractions [69]. 

#### 4.3.5. Antagonism of Hypothermia and Ptosis Induced by Tetrabenazine

The mice whose rectal temperatures were measured as 36–38 °C received each test sample/reference drug orally 60 min before the tetrabenazine injection (32 mg/kg) intraperitoneally which was dissolved in 0.1 M tartaric acid (pH changed to 6 using NaOH 10%). After 30 min, the mice were put at the center of a disk (diameter of 20 cm) and the degree of palpebral ptosis and the akinesia exhibited by each mouse was assessed within 10 s. The ptosis degree was rated in accordance with the following rating scale: 0 for eyes open; 1 for one-quarter closed; 2 for half closed; 3 for three-quarters closed; and 4 for completely closed [36]. The mice were not accepted akinetic if they gave at least one of the following responses: Walking to the edge of the disk and looking over the side, moving 180° in place, 90° head movement followed by a 45° movement in the opposite direction [34]. If the animal had locomotor activity, sedation was thought to be prevented. The number of mice exhibiting a positive comeback was divided by the number of animals in the group and multiplied by 100 to obtain the percentage of animals exhibiting locomotor activity at the indicated dose. The rectal temperature of each mouse was measured with a thermometer 60 min after the injection of tetrabenazine and the temperature changes were calculated. Four different experimental groups were formed for the evaluation the effect of each extract and six experimental groups were used to assess the activity of the fractions [69]. 

#### 4.3.6. The Inhibitory Activity on the MAO A&B

The effect of extracts, fractions and isolated compounds from MeOH extract on the activity of MAO A and B were investigated in vitro in the brain and liver of the rats. Enzyme sources were prepared according to the method of Hwang (2003) [71] from brain of Sprague–Dawley male rats. The anesthetized rats lost blood with 3.13% sodium citrated syringe from the heart. The brain tissue was obtained from the decapitated brain which was washed with 0.01 M phosphate buffered saline (PBS, pH 7.0), and homogenate at 40 °C for 1 min followed by adding cold 0.25 mM sucrose by nine parts of wet weight of tissue. Centrifuged at 700 g in 40 °C for 20 min. Supernatant was centrifuged at 18,000 g for 20 min immediately. Pellet was suspended in five parts of PBS, and used for crude enzyme preparation. Prepared crude MAO-A (0.5 mL) was added to test tubes with 1.0 mL of test materials. It was incubated in shaking incubator at 37.5 °C for 15 min. As a substrate, 0.5 mL of 1.0 mM serotonin was added and incubated at 37.5 °C for 90 min. To terminate the enzyme action, test tubes were heated at 95 °C water bath for 3 min. and centrifuged at 700 g for 20 min. immediately. Supernatants were poured in prepared Amberlite CG-50 (H+ form) column (0.6 × 4 cm). After washing with distilled water thoroughly (over 40 mL), eluted with 3 mL of 4 N acetic acid, elute was determined of absorbance at 277 nm. Instead of samples, the same volumes of distilled water were added in the control. In the sample controls, the substrates were added on the time of activity termination instead of initiation of action. Each group was performed in duplicate and calculated for the inhibition percentages of samples by proper expression.

## Figures and Tables

**Table 1:** Effects of the extracts, fractions from MeOH extract and pure compounds from *M. myrtifolia* on the forced swimming test.

Material	Dose (mg/kg p.o.)	Duration of Immobility (s) (Mean ± S.E.M.)
Control	-	187.22 ± 6.16
*n*-Hexane extract	100	199.51 ± 7.74
EtOAc extract		173.08 ± 8.13
MeOH extract		115.34 ± 5.12 *
Imipramine	30	109.61 ± 4.23 **
	50	94.26 ± 4.01 ***
Control	-	173.12 ± 5.01
Fr. A	100	192.31 ± 3.96
Fr. B		105.18 ± 3.26 **
Fr. C		98.15 ± 2.07 ***
Fr. D		162.45 ± 2.93
Fr. E		181.26 ± 4.12
Imipramine	30	112.73 ± 2.84 **
	50	93.28 ± 2.85 **
Control	-	191.89 ± 9.68
Rosmarinic acid	25	119.76 ± 6.49 **
Myricetin		118.19 ± 4.62 **
Apigenin		132.15 ± 7.06 **
Naringenin		136.63 ± 6.81 *
Imipramine	30	128.00 ± 5.19 **
	50	97.02 ± 3.74 **

* *p* < 0.05; ** *p* < 0.01; *** *p* < 0.001; S.E.M.: Standard Error of Mean.

**Table 2:** Effects of the extracts, fractions from MeOH extract and pure compounds from *M. myrtifolia* on the tail suspension test.

Material	Dose (mg/kg p.o.)	Duration of Immobility (s) (Mean ± S.E.M.)
Control	-	172.68 ± 6.14
*n*-Hexane extract	100	191.41 ± 11.23
EtOAc extract		154.80 ± 7.59
MeOH extract		111.82 ± 5.62 *
Imipramine	30	105.41 ± 3.62 **
	50	91.56 ± 2.48 ***
Control	-	195.75 ± 10.63
Fr. A	100	182.03 ± 9.21
Fr. B		123.92 ± 5.04 *
Fr. C		131.37 ± 6.89
Fr. D		184.91 ± 10.17
Fr. E		197.66 ± 12.28
Imipramine	30	108.82 ± 4.90 **
	50	95.42 ± 3.20 ***
Control	-	220.49 ± 17.04
Rosmarinic acid	25	119.83 ± 11.91 *
Myricetin		189.72 ± 15.26
Apigenin		176.17 ± 12.03
Naringenin		142.58 ± 14.45
Imipramine	30	101.64 ± 4.77 **
	50	84.90 ± 2.49 ***

* *p* < 0.05; ** *p* < 0.01; *** *p* < 0.001; S.E.M.: Standard Error of Mean.

**Table 3:** Effects of the extracts, fractions from MeOH extract and pure compounds from *M. myrtifolia* on antagonism of tetrabenazine-induced ptosis and hypothermia.

Material	Dose (mg/kg p.o.)	Ptosis Mean Score (30 min) (Mean ± S.E.M.)	Mean Decrease in Rectal Temperature (°C) (Mean ± S.E.M.)
Control	-	3.19 ± 0.63	4.88 ± 0.32
*n*-Hexane extract	100	3.24 ± 0.52	4.91 ± 0.74
EtOAc extract		2.71 ± 0.49	4.07 ± 0.52
MeOH extract		2.13 ± 0.21 *	2.46 ± 0.21 *
Fluoxetine	25	0.00 ± 0.00 ***	0.43 ± 0.17 **
Control	-	4.16 ± 0.92	5.93 ± 0.48
Fr. A	100	3.96 ± 0.38	4.82 ± 0.54
Fr. B		2.34 ± 0.21 *	2.82 ± 0.10 *
Fr. C		2.91 ± 0.18 *	3.06 ± 0.27 *
Fr. D		4.29 ± 0.63	5.11 ± 0.82
Fr. E		4.57 ± 0.51	6.12 ± 0.43
Fluoxetine	25	0.00 ± 0.00 ***	0.49 ± 0.11 ***
Control	-	3.95 ± 0.83	6.18 ± 1.07
Rosmarinic acid	25	2.18 ± 0.66 *	3.11 ± 0.74 *
Myricetin		2.53 ± 0.48 *	2.49 ± 0.42 *
Apigenin		2.31 ± 0.45 *	2.62 ± 0.58 *
Naringenin		3.22 ± 0.94	3.34 ± 0.87
Fluoxetine		0.00 ± 0.00 ***	0.36 ± 0.15 ***

* *p* < 0.05; ** *p* < 0.01; *** *p* < 0.001; S.E.M.: Standard Error of Mean.

**Table 4:** Effects of the extracts, fractions from MeOH extract and pure compounds from *M. myrtifolia* on the MAO inhibition assay.

Material	IC_50_ (mg/mL)	
	MAO-A	MAO-B
Control	>10	>10
*n*-Hexane extract	>10	7.3
EtOAc extract	>10	8.9
MeOH extract	4.7	1.4
Fr. A	>10	>10
Fr. B	5.9	4.8
Fr. C	4.1	2.2
Fr. D	>10	>10
Fr. E	>10	>10
Rosmarinic acid	6.5	5.3
Myricetin	4.4	2.7
Apigenin	3.1	1.6
Naringenin	5.9	1.9
Caffeine	0.2	0.5
